# On and Off: Epigenetic Regulation of *C. albicans* Morphological Switches

**DOI:** 10.3390/pathogens10111463

**Published:** 2021-11-11

**Authors:** Elise Iracane, Samuel Vega-Estévez, Alessia Buscaino

**Affiliations:** Kent Fungal Group, School of Biosciences, University of Kent, Canterbury, CT2 7NJ, UK; E.Iracane@kent.ac.uk (E.I.); S.Vega-Estevez@kent.ac.uk (S.V.-E.)

**Keywords:** *Candida albicans*, epigenetic, yeast, chromatin, biofilm, hyphae

## Abstract

The human fungal pathogen *Candida albicans* is a dimorphic opportunistic pathogen that colonises most of the human population without creating any harm. However, this fungus can also cause life-threatening infections in immunocompromised individuals. The ability to successfully colonise different host niches is critical for establishing infections and pathogenesis. *C. albicans* can live and divide in various morphological forms critical for its survival in the host. Indeed, *C. albicans* can grow as both yeast and hyphae and can form biofilms containing hyphae. The transcriptional regulatory network governing the switching between these different forms is complex but well understood. In contrast, non-DNA based epigenetic modulation is emerging as a crucial but still poorly studied regulatory mechanism of morphological transition. This review explores our current understanding of chromatin-mediated epigenetic regulation of the yeast to hyphae switch and biofilm formation. We highlight how modification of chromatin structure and non-coding RNAs contribute to these morphological transitions.

## 1. Introduction

Epigenetics is a popular term first defined by Conrad Waddington in the early 1940s as "the process by which the genotype brings the phenotype into being" [1]. Since then, the meaning of epigenetics has significantly changed. Arthur Riggs defined epigenetics as the study of mitotically and/or meiotically heritable changes in the gene function that are not explained by changes in the DNA sequence [2]. Riggs’ definition focuses on heritability: the ability of an epigenetic mark to be passed to subsequent generations of cells and/or organisms. There is no doubt that heritable epigenetics is an important regulatory mechanism. However, this definition excludes many important, not heritable mechanisms often labelled as "epigenetic". For example, post-translation modifications of histone proteins and their effect on gene expression are often described as an epigenetic regulatory mechanism. However, chromatin marks are, in the majority of the cases, transient and not heritable. Likewise, Riggs’ definition excludes the role of non-coding RNAs (ncRNAs) in transcription and other DNA-based organisms. To overcome this conundrum, Adrian Bird redefined epigenetics as "the structural adaptation of chromosomal regions to register, signal or perpetuate altered activity states" [3]. This definition focuses on changes in gene function that are independent of changes in the underlying DNA sequence. Importantly, these changes can be heritable or not. Epigenetic regulatory mechanisms include changes in gene expression and chromosome function triggered by chromatin modification, chromatin remodelling and ncRNAs activity [2,3,4,5,6,7,8]. In this review, we will adopt Adrian Bird’s definition.

Human fungal pathogens are microbial organisms that kill more than 1.5 million people annually and reduce the quality of life of >1 billion people [9]. Additionally, the recent staggering escalation in the number of invasive fungal infections and the emergence of antifungal drug resistance poses an ever-increasing threat to human health. Fungal pathogens grow in association with their host, and establishing how these organisms adapt to hostile host environments is key to understanding how they cause life-threatening infections and develop resistance to antifungal drugs. 

Chief among human fungal pathogens is *Candida albicans,* a CTG(Ser1)-clade organism in which the CTG codon is translated as serine rather than leucine [10,11]. *C. albicans* colonises almost every organ in the human body, and therefore, it is exposed to rapid environmental changes [12]. Indeed, *C. albicans* is a harmless commensal yeast found in the skin, gut, oral cavity and mucosa [13]. However, this fungal pathogen can become virulent, establishing an extensive range of mucosal and systemic infections. For example, *C. albicans* can cause vulvovaginal candidiasis (VVC), an infection estimated to afflict 75% of all women at least once in their lifetime [14] or candidiasis, systemic infections that can be life-threatening in immunocompromised individuals and are associated with high mortality rates (up to 50%) [9]. Phenotypic plasticity is a critical regulatory mechanism that drives rapid adaptation to hostile host environments. Indeed, environmental changes can induce dramatic morphological changes, and phenotypic switches are critical host adaptation and virulence drivers. For example, *C. albicans* can grow as a single rounded yeast cell or as multicellular hyphae. Yeast cells are critical for host colonisation, early infection and dissemination, while hyphae facilitate tissue invasion and damage [15,16]. Filamentous cells are also crucial for biofilm formation, a highly organised structure that confers resistance to antimicrobial therapies and the host immune response [17]. *C. albicans* cells can also switch between a white and opaque state. White and opaque cells have different appearances, gene expression profiles and mating behaviours [18].

Epigenetic regulatory mechanisms are emerging as essential modulators of *C. albicans’* phenotypic plasticity. Indeed, epigenetic regulation can sense environmental changes leading to the rapid and reversible modulation of gene expression and adaptation to hostile environments. Recently, Qasim et al. [19] reviewed the role of epigenetics in the white–opaque switch extensively. This review will discuss the contribution of epigenetics to *C. albicans* phenotypic plasticity by focusing on the gene-regulation changes in the yeast -hyphae switch and biofilm formation.

## 2. *C. albicans’* Chromatin Structure: The Basics

**Figure 1:**
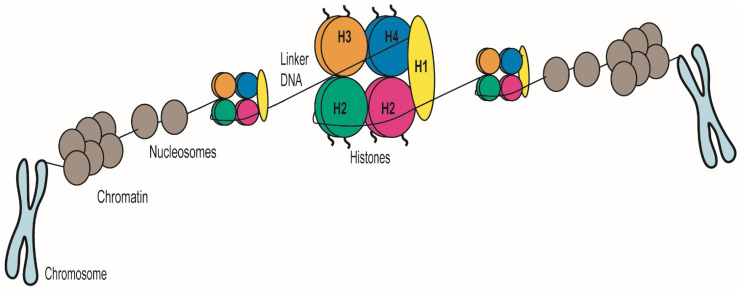
Chromatin organisation and compaction in eukaryotes. Chromatin, a DNA–protein complex, forms chromosomes within the nucleus of eukaryotic cells. The central unit of chromatin is the nucleosome composed of a histone octamer and DNA. Histone H1 promotes further chromatin folding in some eukaryotes.

In eukaryotes, DNA is packed around specific histone proteins within the nucleus to form a compact structure called chromatin. The basic unit of chromatin is the nucleosome, formed by 147 base pairs (bp) of DNA wrapped around an octamer of histones. This octamer is composed of two dimers of the histones H2A–H2B and the histone tetramer (H3)_2_(H4)_2_. Nucleosomes are organised into arrays that are further packaged by histone H1, promoting chromatin folding into compact fibres [20] (Figure 1). The diploid *C. albicans* genome contains two homologous pairs of divergently transcribed histones H2A (*HTA1* (orf19.6924)) and H2B (*HTB1* (orf19.6925)), as well as histone H3 (*HHT2* (orf19.1853) and *HHT21* (orf19.1061)) and H4 (*HHF1* (orf19.1059) and *HHF2* (orf19.1854)) genes. A putative histone H1 (*HHO1* (orf19.5137.1)) can also be identified [21]. Although histones are slow-evolving proteins, variability in histone proteins has been documented in most eukaryotes and histone variants play critical biological roles [22,23,24]. For example, the histone H3 variant, CENP-A^Cse4^, epigenetically defines centromeres in each chromosome. *C. albicans* CENP-A^Cse4^ marks regional centromeres associated with its eight chromosomes [25].

**Figure 2:**
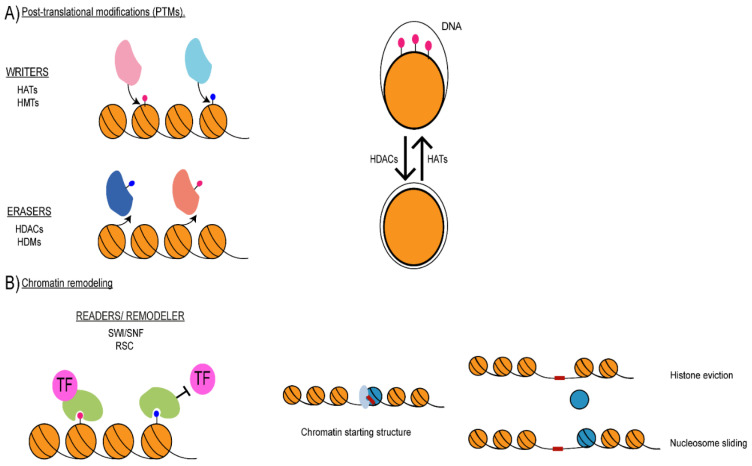

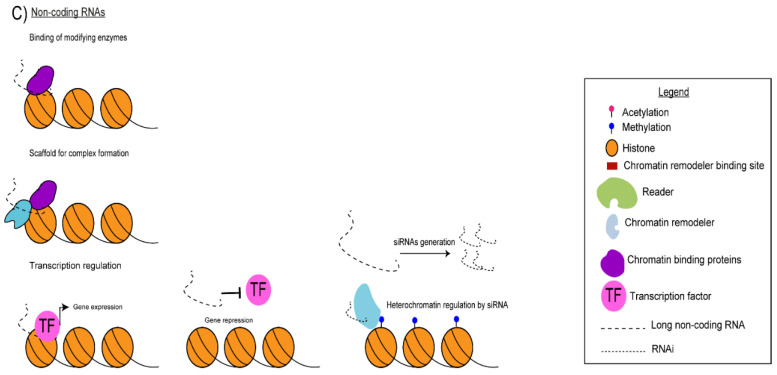
Mechanisms of modulation in chromatin structures. (**A**) Left: schematics of chromatin writers and erasers: writers, such as HATs and HMTs, add epigenetic marks to histone proteins, while erasers, such as HDACs and HDMs, remove epigenetic marks from histone proteins. Right: schematic of how post-translation modifications, such as histone acetylation, can affect DNA-histone interactions. (**B**) Modes of action of readers and chromatin remodelers. Left: reader proteins, components of chromatin remodelling complex, bind modified histone tails recruiting or blocking transcription factors. Right: schematics of chromatin remodellers’ activity, including histone eviction and nucleosome sliding. (**C**) Mode of action of non-coding RNAs (ncRNAs). Top: ncRNAs can recruit modifiers to chromatin or act as a scaffold promoting the formation of protein complexes. Middle: ncRNAs can activate or repress transcription. ncRNAs can also be processed into siRNAs by the RNAi machinery. siRNAs can seed heterochromatin formation. HAT: histone acetyltransferase; HMT: histone methyltransferase; HDAC: histone deacetylase; HDM: histone demethylase; RSC: remodels the structure of chromatin.

Chromatin allows the packaging of DNA into a compact structure that can fit inside the nucleus while permitting efficient accessibility to DNA-binding proteins. However, chromatin is also an obstacle to all DNA-templated biological processes, including transcription, replication, recombination and repair [26]. Consequently, changes in chromatin structure can have a profound impact on nuclear processes, and chromatin is a crucial regulator of DNA-based activities. For example, chromatin can be assembled into two functionally and structurally different chromatin structures. Gene-rich regions and non-repetitive DNA are assembled into euchromatin, an open chromatin state that is permissive to transcription. In contrast, heterochromatin is a transcriptionally silent chromatin state that is associated with gene-poor and repetitive regions of the genome [27]. Chromatin structure can be modulated by three distinct mechanisms: (i) post-translation modification of histone proteins, (ii) chromatin remodelling and (iii) ncRNAs (Figure 2).

## 3. Histone Post-Translational Modifications

Histone proteins are formed by a globular core and unstructured basic amino-terminal tails that can be post-translationally modified. The most common post-translational modifications (PTMs), also known as histone marks, include methylation, acetylation, ubiquitination, ADP-ribosylation and the sumoylation of lysine (K) residues; the methylation of arginine (R) residues and the phosphorylation of serine (S) and threonine (T) residues. In addition, the same amino acid can be affected by multiple modifications (i.e., mono, di- or tri-methylated) [28].

**Figure 3:**
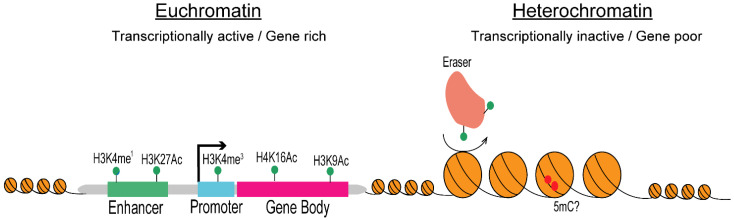
Histone modifications and transcriptional activity in *C, albicans*. (**Left**): schematic of histone modifications associated with active genes that are assembled into euchromatin. Coding regions (gene body) and regulatory regions (enhancer and promoter) are shown. (**Right**): schematic of chromatin marks associated with gene-poor heterochromatic regions.

Histone marks are differentially associated with euchromatin and heterochromatin regions. At euchromatic transcriptionally active regions, genes promoters are assembled into a chromatin state containing acetylated histones that are tri-methylated on H3K4 (H3K4me^3^), while histone H3 methylated on K36 (H3K36me) is found at gene bodies [26]. Likewise, enhancers and super-enhancers are marked by the mono-methylation of histone H3 on K4 (H3K4me^1^) and the acetylation of histone H3 on K27 (H3K27Ac) [29,30]. Genome-wide chromatin profiling demonstrates that the *C.* *albicans* transcriptionally active genome is packaged into canonical euchromatin, where gene promoters of active genes are enriched in H3K4me^3^ while gene bodies are marked byacetylated histone H3 (H3K9Ac) and H4 (H4K16Ac) (Figure 3) [31]. In many eukaryotic organisms heterochromatic regions are enriched in repressive histone marks, such as the methylation of K9 on histone H3 (H3K9me) or the methylation of K27 on histone H3 (H3K27me). Furthermore, high levels of DNA methylation on position five of cytosines (5mC) are associated with heterochromatin [26,32]. Similarly to *S. cerevisiae*, *C. albicans* is devoid of H3K9me and H3K27me [33]. Although 5mC mark has been detected in *C. albicans*, it is unclear whether DNA methylation is associated with heterochromatic regions in this organism [34]. Instead, chromatin profiling studies have demonstrated that *C. albicans* heterochromatic regions are characterised by low levels of both histone acetylation and methylation [31] (Figure 3).

The post-translation modification of histone proteins is a dynamic and reversible process catalysed by "writer" and "eraser" enzymes that add and remove epigenetic marks (Figure 2). For example, the additions of acetyl groups to histone tails are carried out by histone acetyltransferases (HATs), whereas their removal is conducted by histone deacetylases (HDACs); methylation marks are added by histone methyltransferases (HMTs) and removed by histone demethylases (HDMs). Additionally, HAT and HDAC can also catalyse the addition and removal of acyl groups different from those in acetylation, such as crotonyl, succinyl, β-hydroxybutyryl, and propionyl [35]. The primary histone modifiers found in *C. albicans* and their orthologs found in *S. cerevisiae*, *S. pombe* and humans are listed in Table 1.

Histone marks alter chromatin architecture and its function via two main distinct mechanisms. Firstly, PMTs can alter histone-DNA interaction modulating higher-order chromatin structure and affecting gene expression and regulation [39]. For example, histone acetylation reduces the net positive charge of histone tails, and therefore, will weaken histone–DNA interaction, resulting in an open chromatin conformation that is permissive to transcription (Figure 2A) [39]. Histone crotonylation activates transcription more potently than histone acetylation [40]. This is because the crotonyl group is more hydrophobic and rigid than acetyl groups, disrupting histone-DNA interactions [40]. Histone marks can also be recognised by "reader" proteins, which can influence chromatin dynamics and function via promoting or blocking the recruitment of transcription factors and/or other chromatin-modifying factors (Figure 2B) [41]. For example, bromodomain-containing proteins specifically bind acetylated histones, chromodomain containing proteins recognise specific methylation marks and the YEATS domain recognises the crotonyl marks [42].

## 4. Chromatin Remodelling Regulates Gene Expression and Chromatin Structure

Nucleosomes deposited on DNA can be a physical barrier, reducing chromatin accessibility and gene expression. Chromatin remodelling is the regulatory process that changes the interactions between DNA and histone proteins leading to complete or partial disassembly of the nucleosomes (histone eviction) or nucleosome reposition (nucleosome sliding) [43] (Figure 2C). Chromatin remodelling is catalysed by ATP-dependent multi-subunit protein complexes known as chromatin remodelers [43]. ATP-dependent chromatin remodelers belong to four subfamilies: switch/sucrose non-fermentable (SWI/SNF), imitation switch (ISWI), chromodomain helicase DNA-binding (CHD/NuRD/Mi-2) and inositol-requiring 80 (INO80) [44]. Among those, the SWI/SNF subfamily is the primary remodeler catalysing nucleosome sliding and eviction. Initially identified in budding yeast, SWI/SNF complexes are highly conserved across eukaryotes [45]. The SWI/SNF complex can be targeted to acetylated transcriptionally active chromatin, as it can bind acetylated histones (and non-histone proteins) through a bromodomain subunit [44]. Therefore, SWI/SNF activity generally correlates with transcriptional activation even if the complex has also been linked to transcriptional repression [46,47,48,49,50].

Different yeast species contain a second remodelling complex similar to SWI/SNF, the RSC (remodels the structure of chromatin) complex [44]. This complex is essential for survival in *S. cerevisiae,* although it is not required for growth in *S. pombe* [51]. The RSC complex binds promoters and intergenic regions and is specifically recruited to RNA polymerase II to tune gene transcription [52]. Four chromatin remodeler catalytic subunits have been described in *C. albicans*: *STH1*, *SNF2*, *SWR1* and *SWI1*. Sth1 is the catalytic subunit of the RSC complex, which in *C. albicans* is composed of a total of 13 subunits, including two CTG (Ser1)-clade-specific (Nri1 and Nri2) [53]. Snf2 and Swi1 are catalytic subunits of the SWI/SNF complex [54], and Swr1 is the major subunit of the SWR1 complex [55]. 

## 5. Non-Coding Transcription and Non-Coding RNAs 

**Figure 4:**
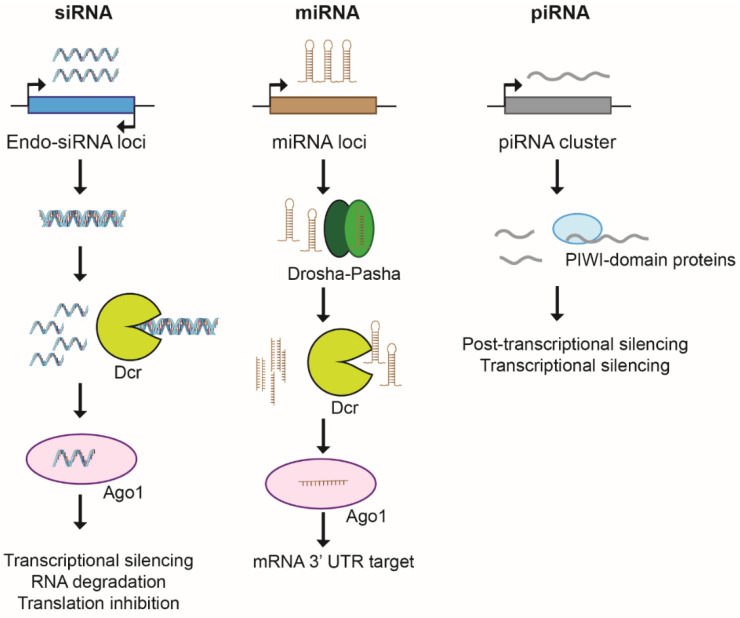
RNA interference pathways. In the siRNA pathway, a precursor dsRNA is processed into a siRNA duplex by dicer (Dcr), and one strand of the duplex is loaded into argonaute (Ago1), which targets it against complementary RNAs for inactivation. In the miRNA pathway, loop dsRNA precursors are processed, first, in the nucleus by drosha–pasha and later in the cytoplasm by dicer to produce miRNAs. These are loaded into an Ago and targeted to the 3′-UTR region of the target mRNA to block translation. In the piRNA pathway, a single transcript is generated from a piRNA cluster and processed into piRNAs by PIWI-domain-containing proteins that silence their targets post-transcriptionally in the cytoplasm through piRNA direct cleavage or, transcriptionally, in the nucleus via heterochromatin assembly.

Large fractions of eukaryotic genomes are extensively transcribed but not translated into functional proteins. The act of non-coding transcription and its associated histone modifications and changes in nucleosome density can interfere with the activity of nearby genes [56]. However, ncRNAs can also regulate gene expression by interacting with DNA, RNA and proteins and modulating chromatin structure [57]. Finally, ncRNAs can be processed into small silencing RNAs by RNA interference (RNAi) machinery. The RNAse III-like enzyme dicer (Dcr) and the PIWI domain-containing protein Argonaute (Ago) are at the core of the RNAi machinery and responsible for the generation of the three major branches of small ncRNAs—short interference RNAs (siRNA), micro RNAs (miRNAs) and PIWI-interacting RNAs (piRNAs)—that differ in their biogenesis and mechanisms of action (Figure 4). 

In the siRNA pathway, RNAi is triggered by a dsRNA precursor that can arise endogenously by transcription of repetitive DNA and by convergent transcription. This precursor dsRNA is processed into a 20–24-nucleotide (nt) siRNA duplex by Dcr [58]. One strand of the duplex is loaded into Ago, an effector complex. Ago uses base-pairing interaction to target cognate RNAs for inactivation. siRNA-mediated silencing can be co-transcriptional by seeding the assembly of repressive heterochromatin or post-transcriptional RNA cleavage using Ago-slicer endonuclease activity. siRNA-directed heterochromatin assembly has been best described in the fission yeast *S. pombe*, wherein RNAi machinery triggers the formation of transcriptionally silenced hypoacetylated chromatin, methylated on lysine 9 of histone H3 (H3K9) [59]. In the miRNA pathway, short stem-loop dsRNA precursors are pre-processed by a nuclear RNAse III complex (Drosha–Pasha) before final processing into miRNAs in the cytoplasm by Dcr. miRNAs are loaded into an Ago-containing protein complex and targeted to the 3′ untranslated region (3′-UTR) of the target mRNA blocking its translation [60]. The piRNA pathway silences transposable elements in the germline of many animal species [61]. In contrast to the other pathways, piRNAs are not generated by dsRNAs precursors and their biogenesis is independent of Dcr [62]. A single transcript is generated from a piRNA cluster and processed into piRNAs by PIWI-domain containing proteins. The piRNA pathway controls transposons through several distinct but interlinked mechanisms. Whereas cytoplasmic PIWI proteins silence their targets post-transcriptionally through piRNA direct cleavage, nuclear Piwi–piRNA complexes function at the transcriptional level via heterochromatin assembly [63]. Although we still know very little about the nature and the putative function of *C. albicans* non-coding RNAs, RNA profiling analyses have identified many non-coding transcripts whose expression differ under distinct growth conditions [64].

Furthermore, it has been shown that *C. albicans* contains active RNAi machinery in vitro and in a heterologous yeast system. CaDcr1 is a non-canonical enzyme that can generate small RNAs and catalyse the 35S ribosomal RNA [65]. Future studies will establish the impact of non-coding RNAs in *C. albicans* biology.

## 6. Chromatin-Mediated Regulation of the Yeast to Hypha Morphological Switch

Modulation of the yeast to hypha morphological transition relies on a complex interplay of a transcriptional regulator and chromatin modifiers [66,67]. Hyphal growth can be divided into two different stages: initiation and maintenance. In yeast cells, the transcriptional repressors Nrg1 and Tup1 inhibit hyphal morphogenesis by blocking the expression of a subset of filament-specific genes [68,69]. During the initiation stage, Nrg1 protein levels decrease sharply, and the Nrg1-mediated repression is cleared. After that, during the maintenance phase, Nrg1 protein levels recover rapidly, but Nrg1 binding to promoters of hypha-specific genes is inhibited [70,71].

**Figure 5:**
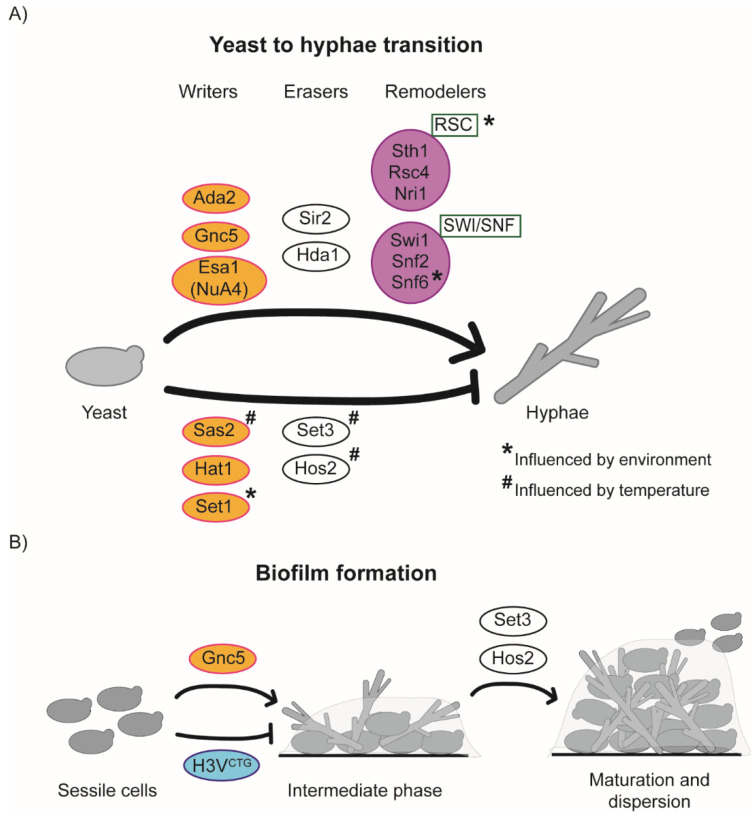
Influence of chromatin modifiers and remodelers on (**A**) the yeast–hyphae transition and (**B**) biofilm formation in *C. albicans*. Proteins promoting the yeast-to-hyphae transition or biofilm formation are represented on top. Proteins repressing the yeast-to-hyphae transition or biofilm formation are represented below. Proteins influenced by the environmental conditions or temperature are represented with (*) or (#) respectively. Writers, erasers, remodelers and histone variants are represented in orange, white, magenta and blue bubbles, respectively.

Hyphal growth is induced by a broad range of environmental and host factors, including serum, nutrient starvation, hypoxia and high CO_2_ concentration [72,73]. These different host signals are integrated by redundant sensing pathways that modulate the activity of transcriptional regulators (such as Chp1, Egf1 and Flo8), resulting in the transcriptional upregulation of hundreds of genes such as genes encoding for cell wall proteins, adhesins and secreted aspartyl proteinases (SAP) [74]. Chromatin modifiers are emerging as important regulators of the yeast-to-hypha transcriptional programme [75,76,77] (Figure 5A). For example, the concerted and opposite activities of the NuA4 HAT complex and the Hda1 HDAC are necessary for the initiation–maintenance transition and for activating the hyphal-specific transcriptional programme. Upon hyphae induction, the NuA4 complex is recruited to the promoters of hyphae-specific genes. Dynamic acetylation of histone H4 and the NuA4 components Yng2 have been proposed to be necessary for NuA4-dependent hypha induction [75,78]. Consequently, deletion of the *ESA1* gene, encoding for the catalytic subunit of the NuA4 complex, hinders filamentous growth [75]. The HDAC Hda1 promotes hypha maintenance by deacetylating Yng2, and this modification is critical to sustaining hyphal maintenance blocking Nrg1 binding to hyphae-specific promoters in response to serum or nutrient limitation [79]. Importantly, HDA1 is not required for hyphae maintenance or elongation in hypoxia or the presence of elevated CO_2_, demonstrating the complexity of the hyphae regulatory programme [80,81]. 

Several other histone modifiers are important for the yeast–hyphae switch. For example, the HAT Gcn5 is a positive regulator of hyphal growth, while the HATs Sas2 and Hat1 are negative regulators of hyphae formation [75,82,83]. The Set3/Hos2 histone deacetylase complex negatively regulates the yeast-to-hyphae switch by modulating the kinetics of the filamentous transcriptional programme [76,84]. Furthermore, the catalytic activity of the HDAC Sir2 modulates hyphae formation, as the number of hyphae is reduced in a *C. albicans* strain expressing catalytic inactive Sir2 [77]. 

It is largely unknown how chromatin modifiers modulate the yeast-to-hyphae switch, as the critical substrates necessary for this morphological transition have not been identified yet. Identifying these substrates will be essential to unveil the role of protein post-translation modifications in filamentous growth as chromatin modifiers modify histones and non-histones proteins [85,86]. Furthermore, histone crotonylation is emerging as a crucial post-translation modification regulating *C. albicans* filamentous growth [87]. As HATs can catalyse both acetylation and crotonylation, it will be essential to dissect which modifications are the key regulators of filamentous growth. 

Several studies demonstrate that chromatin remodelling controls the yeast-to-hyphae transition. Indeed, the *C. albicans* SWI/SNF and RSC chromatin-remodelling complexes are required for filamentation growth [54,88,89]. However, the molecular mechanism(s) of the SWI/SNF-mediated regulation of hypha formation is still unclear. Indeed, it has been shown that, upon hyphal induction, the SWI/SNF catalytic subunit Snf2 binds the promoters of the hyphae-specific genes *HWP1*, *ALS3* and *ECE1* [78]. This observation suggests that SWI/SNF directly controls the filamentous transcriptional programme by chromatin remodelling of hyphae-specific genes. However, genome-wide chromatin profiling of a different SWI/SNF component, Snf6, did not detect any specific interaction with hyphae-specific genes (including *HWP1*, *ALS3* and *ECE1*). Furthermore, RNA sequencing analyses of wild type (WT) and *SNF6* deletion strains suggest that SWI/SNF is a general transcriptional regulator in both yeast and hyphal cells and that SWI/SNF controls filamentation indirectly [88]. Future studies will determine whether Snf2 plays a role in hyphae formation independently of other SWI/SNF components. 

Although the role of non-coding RNAs in the modulation of hyphal transition is largely unexplored, genome-wide gene-expression profiling studies have identified several novel ncRNAs specifically expressed in hyphae-inducing growth conditions [64,90]. It is still unknown whether these ncRNAs have a function, but it is interesting to note that some of these non-coding RNAs have expression profiles similar to the expression profile of hyphae-specific genes. In the future, it will be essential to determine the function of these ncRNAs.

## 7. Chromatin-Mediated Regulation of the Planktonic-Biofilm Transition

*C. albicans* biofilm consists of a layer of yeast cells overlaid by filamentous hyphal and pseudo-hyphal cells surrounded by an extracellular matrix formed by polysaccharides and proteins. The formation of biofilms is a multi-step process consisting of four stages: (1) the adherence and colonisation of yeast cells to the surface, (2) yeast cell proliferation forming the basal layer, (3) the growth of hyphae and pseudo-hyphae with the formation of the extracellular matrix and complex three-dimensional architecture, (4) the dissemination of progeny biofilm cells to seed new sites [91]. Seven master regulators (Bcr1, Brg1, Efg1, Flo8, Ndt80, Tec1 and Rob1) are critical for normal biofilm formation in vivo and in vitro [92,93]. Of these seven regulators, Bcr1, Efg1 and Ndt80 are important modulators of biofilm formation in non-albicans *Candida* species that are evolutionarily distant from *C. albicans* [94]. The biofilm master regulators are transcriptional regulators controlling the expression of thousands of genes expressed differentially between yeast and biofilm cells [95].

An increasing body of evidence suggests that chromatin modifiers and chromatin remodelling regulate different stages of biofilm formation (Figure 5B). Firstly, a specific chromatin state, marked by the histone H3 variant H3V^CTG^ (*ORF19.6791)*, acts as a negative regulator of biofilm formation in planktonic cells [96]. H3V^CTG^ contains three variant amino acids (Ser31, Thr32 and Thr80) replaced by Val31, Ser32 and Ser80. Val31 and Ser32 are essential for the variant function. H3V^CTG^ binds promoters of biofilm-related genes in planktonic cells, but it does not mark these gene promoters in biofilm-inducing growth conditions. Additionally, H3V^CTG^ mutant strains produce more robust biofilms than WT cells in vivo and in vitro, suggesting that H3V^CTG^ represses biofilm formation [96]. The role of H3V^CTG^ in other CTG-clade yeast species is unknown. H3V^CTG^ likely regulates biological processes distinct from biofilm formation as this histone variant is expressed in CTG-clade organisms such as *Scheffersomyces stipitis* and *Debaryomyces hansenii* that do not form biofilm under several biofilm-inducing conditions [94,96].

Hyphae formation is important in the biofilm process. Therefore, it is likely that chromatin modifiers regulating filamentous growth are also required for biofilm maturation. Accordingly, deletion of the HAT *GCN5* leads to a strong decrease of adhesion and a dysregulation of Als1-mediated adhesion, which hints at the role of Gcn5 in biofilm establishment [84]. Additionally, it has been shown that chromatin-mediated transcriptional regulation is important for regulating biofilm dispersal. Indeed, the HDAC Set3/Hos2 is a positive regulator of biofilm dispersal. *C. albicans* strain deleted for the *SET3* gene are hyper filamentous and have a reduced number of yeast cells leading to a reduced biofilm dispersal [84].

It is still unknown whether non-coding RNAs regulate biofilm formation. However, ncRNAs might play an crucial regulatory role in biofilm formation as specific ncRNAs are differentially expressed in biofilm cells compared to planktonic cells [64]. 

## 8. Conclusions

**Figure 6:**
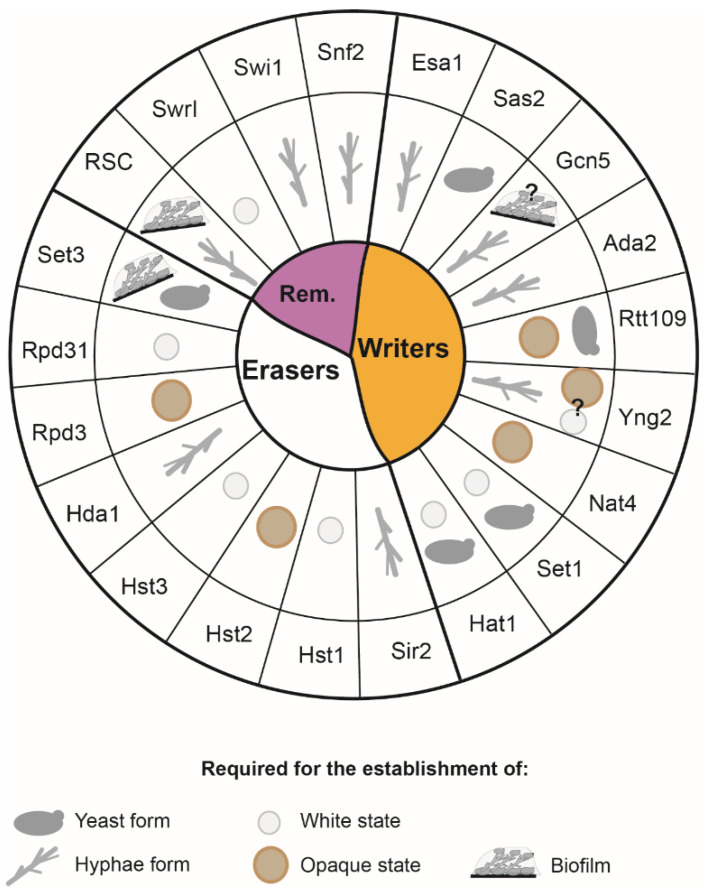
Summary of the involvement of the main erasers, writers and remodelers (Rem.) in the yeast–hyphae transition, the white–opaque switch and biofilm formation. The question mark indicates a possible involvement not confirmed yet.

An increasing body of evidence demonstrates that chromatin modifiers and chromatin remodelers modulate the gene expression programmes associated with the yeast to hyphae switch and with biofilm formation, two interconnected processes playing important roles for host adaption and pathogenesis, as well as the white-opaque switch (Figure 6). Despite the emerging central role of chromatin-mediated regulation in controlling *C. albicans* biology, our understanding of these regulatory processes is still in its infancy. This is because we lack the fundamental knowledge and understanding of how chromatin structures change in different host hostile environments and whether chromatin modulation differs among *C. albicans* clinical isolates. To start filling this gap in knowledge, chromatin profiling of different *C. albicans* morphological forms should be performed. Similarly, histone and non-histone substrates of chromatin modifiers should be identified using biochemical approaches. In addition, it will be exciting to dissect the role of non-coding RNAs and the RNAi machinery to *C. albicans* morphological switches. 

## Figures and Tables

**Table 1:** *Candida albicans* histone modifiers and RNA interference actors and their orthologs in *S. cerevisiae*, *S. pombe* and humans.

	*C. albicans* Gene and Known Function Candida Genome [21]	*S. cerevisiae* Ortholog SGD [36]	*S. pombe* Ortholog Pombase [37]	Human Ortholog Alliance of Genome Resources [38]
**Histone Acetyltransferase**	*ESA1* (orf19.5416) NuA4 HAT complex acts on H4K5, H4K12	*ESA1* (YOR244W) *(alias: TAS1, KAT5)*	*MST1 (SPAC637.12c)*	*TIP60* (*alias: KAT5*)
	*SAS2* (orf19.2087) SAS HAT complex acts on H4K16	*SAS2* (YMR127C) *(alias: KAT8)*	*MST2 (SPAC17G8.13c)*	
	*GCN5* (orf19.705) SAGA/ADA complex	*GCN5* (YGR252W) *(alias: ADA4, SW19, AAS104, KAT2)*	*GCN5 (SPAC1952.05)*	*KAT2B* *(alias: CAF)*
				*KAT2A* (*alias: GCN5*)
	*ADA2* (orf19.2331) SAGA/ADA complex acts on H3K9	*ADA2* (YDR448W) *(alias: SWI8)*	*ADA2* (*SPCC24B10.08c*)	*TADA2B* (*alias: ADA2B*)
				TADA2A *(alias: ADA2A)*
	*RTT109* (orf19.7491) acts on H3K56	*RTT109* (YLL002W) *(alias: KIM2, REM50, KAT11)*	rtt109 (SPBC342.06c)	No ortholog
	*YNG2* (orf19.878) (*alias: NBN1*) NuA4 HAT complex acts on nucleosomal H4	*YNG2* (YHR090C) *(alias: EAF4, NBN1)*	*png1 (SPAC3G9.08)*	*ING3* *(alias: Eaf4, ING2)*
				*ING2* ± (*alias: ING1L*)
				*ING4* ±
				*ING5* ±
	*NAT4* (orf19.4664)	*NAT4* (YMR069W)	*naa40* (*SPCC825.04c*)	*NAA40* (*alias: NAT11, PATT1*)
	*HAT1* (orf19.779)	*HAT1* (YPL001W) *(alias: KAT1)*	*hat1 (SPAC139.06)*	*HAT1* (*alias: KAT1*)
	*SAS3 (orf19.2540)*	*SAS3 (YBL052C)* (*alias: KAT6*)	*MST2 (SPAC17G8.13c)*	*KAT7* (*alias: HBO1, MYST-2*)
				*KAT6A* ± (*alias: MYST-3*)
				*KAT6B* ± (*alias: MYST-4*)
				*KAT8* ± (*alias: MYST-1*)
**Histone Deacetylase**	*HDA1* (orf19.2606) in a complex with Hda2 and Hda3	*HDA1* (YNL021W)	*clr3* (*SPBC800.03*)	*HDAC10* (*alias: HD10*)
				*HDAC6* (*alias: HD6*)
	*SET3* (*orf19.7221*) SET3 HDAC complex with Hos2, Snt1 and Sif2	*SET3* (YKR029C)	*set3 (SPAC22E12.11c)*	*KMT2E* *
		*SET4* (YJL105W) (*SET3* paralog)		*SETD5* *
	*RPD3 (orf19.2834)*	*RPD3* (YNL330C)	*clr6* (*SPBC36.05c*)	*HDAC1* *(alias: KDAC1, RPD3)*
	*RPD31* (orf19.6801)			*HDAC2* (*alias: KDAC2, RPD3*)
	SIR2 (*orf19.1992*) (*alias: SIR21*)	*HST1* (YOL068C) (*SIR2* paralog)	*sir2* (*SPBC16D10.07c*)	*SIRT1* *(alias: SIR2)*
		*SIR2* (YDL042C)		
	*HST1 (orf19.4761)* (*alias: SIR22*)	*HST1* (YOL068C)		
		*SIR2* (YDL042C)		
	*HST2 (orf19.2580)*	*HST2 (YPL015C)*	*hst2* (SPCC132.02)	*SIRT3*
				*SIRT2*
	*HST3* (orf19.1934) Acts on H3K56	*HST3* (YOR025W)	*hst4* (SPAC1783.04c)	No ortholog
**Histone Methyltransferase**	*SET1* (orf19.6009) Acts on H3K4	*SET1* (YHR119W) *(alias: KMT2)*	*set1* (*SPCC306.04c*)	*SETD1B* (*alias: KMT2G*)
				*SETD1A* (*alias: KMT2F*)
**Histone Demethylase**	*RPH1* (orf19.2743)	*RPH1* (YER169W)	jmj3 (SPBC83.07)	*KDM4A*
				*KDM4B*
				*KDM4C*
				*KDM4D*
				*KDM4E*
**Chromatin** **Remodeler**	*SWR1* (orf19.1871) SWR1 complex	*SWR1* (YDR334W)	swr1 (SPAC11E3.01c)	*SRCAP* (*alias: SWR1*)
	*SWI1* (orf19.5657) SWI/SNF complex	*SWI1* (YPL016W) *(alias: ADR6)*	sol1 (SPBC30B4.04c)	*ARID5A* (*alias: MRF1*)
				*ARID5B* (*alias: MRF2*)
	*SNF2* (orf19.1526) SWI/SNF complex	*SNF2* (YOR290C)	*snf21 (SPAC1250.01)*	*SMARCA2*
				*SMARCA4*
	*STH1* (orf19.239) RSC complex	*STH1* (YIL126W)		*SMARCA2*
				*SMARCA4*
**RNA interference**	*DCR1* (orf19.3796)	No ortholog	*dcr1* (SPCC188.13c)	*DICER1*
	*AGO1* (orf19.2903) RISC complex	No ortholog	*ago1* (SPCC736.11)	*PIWIL1*
				*PIWIL2*
				*PIWIL3*
				*PIWIL4*

(*) human orthologs are histone methyltransferase; (±) *S. pombe* ortholog only.

## Data Availability

No new data were created or analyzed in this study. Data sharing is not applicable to this article.
